# Introduction to Problematics of the Special Issue “Green Biosynthesis of Nanomaterials for Biomedical Applications”

**DOI:** 10.3390/jfb13040307

**Published:** 2022-12-19

**Authors:** Oleg V. Mikhailov, Ekaterina O. Mikhailova

**Affiliations:** 1 Department of Analytical Chemistry, Certification and Management Quality, Kazan National Research Technological University, K. Marx Street 68, 420015 Kazan, Russia; 2 Institute of Innovation Management, Kazan National Research Technological University, K. Marx Street 68, 420015 Kazan, Russia

The concept of “green biosynthesis”, as well as the similar and more general concept of “green synthesis”, is collective and includes very diverse synthetic methods, using products vital for activity from various living organisms—of both plant and animal origin—for chemical compound production. This synthesis variant is an integral part of one of the youngest sections in modern chemical science, namely green chemistry (sustainable chemistry), which, according to the IUPAC definition, is “Design of chemical products and processes that minimize or eliminate the use or generation of substances hazardous to humans, animals, plants, and the environment” [1,2]. This synthesis method is suitable for the production of a wide variety of substances—from elemental metals to organometallic compounds, and from molecular hydrogen to supermolecular and supramolecular systems.

Historically, green chemistry arose and was developed from the many existing ideas and research efforts in the period preceding the last decade of the XX century in the context of increasing attention being paid to pollution and environmental problems and natural resource depletion. The ideological basis of this specific chemistry branch was mainly formed in the second half of the 1990s, and the wider spread of this term meant that it eventually began to prevail over “competing” terms such as “pure” and “stable” chemistry [3,4]. Shortly before the beginning of the XXI century, the 12 principles that should guide the application of green chemistry were presented [3]. The key aspects of these principles, in our opinion, are the following:

- The development of processes to maximize the number of raw materials that enter a product;
- The use of renewable raw materials and energy sources;
- Using safe, environmentally friendly substances, including solvents, whenever possible;
- The design of energy-efficient processes;
- The prevention of waste generation, which is considered an ideal form of waste management.

The last few decades have been marked by the very significant application of “green biosynthesis” to solve various problems related to the nanotechnology field, namely, to obtain various nanoparticles and study the physicochemical processes associated with them. It should be noted here that the analysis of links, including keywords related to green technologies in the main databases, since the appearance of the 12 principles in 1998, shows the scale of work related to this topic [5] (Table 1). Interestingly, the SciFinder and Scopus databases contain fewer references; however, they match the established keywords better. In addition, more than 80% of published works indicate that they are “green”, but no specific comments are given [5]. To imagine the publication activity scale in this area, it is enough to indicate the number of papers devoted to the biosynthesis of elemental silver nanoparticles alone indexed in the Scopus database for the period 1980–2014 and published in the world’s leading scientific publisher’s journals (see the table below). The full publication list on this (very narrow) subject in the Scopus database at the end of 2014 was 20,022 (!!) [6]. However, the publication activity on this topic was only intensified in recent years.

In recent years, it is clear that this publication number has sharply increased (at least one and a half times) over the period 2015–2022. Additionally, there is no doubt that a very significant number of articles devoted to the biosynthesis of elemental silver nanoparticles was published in journals not indexed in Scopus, as well as in other sources of scientific information. Moreover, “green biosynthesis” was used not only in reference to silver nanoparticle synthesis, but also in reference to other, very diverse chemical compounds, which is why the number of articles related to this theme will almost certainly be in six figures. Thus, it is extremely important to study the «green synthesis» specifics and the compounds obtained on its basis and to develop the scientific principles for its further use primarily in the so-called life sciences—for the treatment of different diseases, the creation of new drugs, the fight against a variety of infectious agents, etc.

Although in “green biosynthesis”, living organisms and/or products of their vital activity are used to produce nanoparticles, many chemical reactions take place during this process. The whole set of chemical reactions can be divided into two main categories, namely, reactions that change the oxidation degree of the chemical elements included in the substances participating in the reaction (i.e., starting substances and final products) and reactions that do not change the oxidation degrees of chemical elements. In “green biosynthesis”, chemical transformations in both categories can be realized, but the reactions in the first category, known by the generalized term redox reactions (or redox processes), are the most commonly used here. The vital activity products of living organisms are overwhelmingly reducing agents (i.e., electron donors), whereas substances used to produce target compounds (in particular, nanoparticles of elemental metals), on the contrary, are oxidizers (i.e., electron acceptors). The mechanism of nanoparticle synthesis in the framework of redox processes includes three stages [7]: (1) activation, where a redox process occurs, (2) growth, i.e., the formation of nanoparticles due to heterogeneous nucleation, accompanied by a simultaneous increase in their thermodynamic stability; (3) termination, determining the final shape of nanoparticles. This variant of “green biosynthesis” is very convenient for the production of elemental metal nanoparticles when various products of plant metabolism are used as reducing metal salts and stabilize the resulting nanoparticle reagents [8,9,10]. This method is widely applied for nanoparticle synthesis formed by chemical elements such as silver, gold, and platinum, although they can also be used for the nanoparticle preparation of more active metals (copper, lead, nickel, cobalt, etc.). Equally important is the use of various microorganisms for this purpose. This approach differs in simplicity, cheapness, easy scalability, and “environmental friendliness”, i.e., the safety of using the obtained noble metal nanoparticles in medicine. The absence of toxic effects on the human body, as well as targeting effects, is very important in this regard. The reactions in the second category are more typical in another possible variant of “green biosynthesis”. In this case, products of plant and/or animal origin act as a kind of matrix, within which, chemical transformations (reactions) happen, accompanied by the formation of nanoparticles. As a matrix, such natural high-molecular compounds among polypeptides and polysaccharides such as gelatin, agar, carrageenan, etc., can be used. Nanoparticles obtained via this method, as well as nanomaterials based on them, have already attracted increased attention to their use in biomedical applications and are able to open a new chapter in the treatment of various diseases. Various biological activities make them indispensable assistants in the fight against cancer, pathogenic microorganisms (especially antibiotic-resistant strains), viruses, inflammatory processes, diabetes, as antioxidant agents, as well as drug delivery, and other therapy and diagnostics types. Currently, “green” technologies are considered as the basis for the sustainable development of civilization for future generations. Nevertheless, the “green biosynthesis” of nanoparticles definitely needs to be improved; therefore, the following three problems must be solved:

*The interpretive problem of physical and chemical processes arising within the “green biosynthesis” associated with a wide variety of involved biochemical reagents.* This problem is the most essential; despite significant progress in this research, the nanoparticle biosynthesis mechanism is not fully understood, and accordingly, the controlling possibilities in this process are still very limited. First, it concerns “green biosynthesis”, when redox reactions are realized. Biomolecules capable of metal ion reduction and forming nanoparticles are already known for plant biosynthesis, but the involved compounds’ use as capping agents requires further study, because frequently, these molecules’ roles in the biological properties of potentially used nanoparticles are extremely varied. Using microorganisms as a factory for nanoparticle production requires special condition selection—temperature, pH, and a clear knowledge of the biomolecules involved in this process. In addition, for an adequate interpretation of the “green biosynthesis” process mechanism, it is absolutely necessary to analyze all of these features at the quantitative level, which seems to be an extremely difficult task.

*The problem associated with the purification of the resulting nanoparticles.* Nanoparticles and materials based on them produced by “green biosynthesis”, as a rule, carry a specific memory about their origin prehistory, i.e., they contain a fairly significant number of organic impurities. Sometimes, they can be useful because they contribute to enhancing their biological activity (for example, antibacterial). However, quite often, they can increase the aggregation of nanoparticles, which usually worsens their inherent useful properties. As a result, the problem of removing such undesirable impurities from nanoparticles has become particularly urgent and cannot yet be considered satisfactorily solved. Thus, research with the aim of improving existing and developing new methods for isolating nanoparticles from the systems where they were formed is very important.

*A problem related to the reproducibility of biosynthesis results.* It is possible to control the nanoparticle formation process via chemical and physical methods (with varying reliability) to eventually obtain nanoparticles which are standardized in shape and size. It is much more difficult to achieve this via “green biosynthesis” due, firstly, to the large number of factors affecting the final result, and secondly, the fluctuations associated with them being much more pronounced. Accordingly, the result reproducibility achieved via this synthesis variant is significantly worse than that achieved via chemical and physical methods. For “green biosynthesis”, information regarding the influence of concentration–time and temperature parameters used during the experiment is extremely necessary. However, in most published works, this information is scattered and clearly insufficient for far-reaching conclusions to be drawn. If this problem is solved, it will facilitate standardized preparation production containing nanoparticles, including for commercial purposes. On the other hand, it would be extremely significant to solve this problem, as it would enable the maximum output of a target product to be achieved.

In this way, we hope that this Special Issue will make at least a small contribution to this specific and important branch of modern “green chemistry”. Further research which appears in this direction, with the aim of not only solving the problems outlined above, but also exploring other themes related to nanoparticle production, may serve to increase the popularity of the biological synthesis method among researchers working in the field of nanoscience and nanotechnology.

## Figures and Tables

**Table 1:** Publication scenario of synthesis of silver nanoparticles under standard publishers (1980–2014) (according to data presented in [5]).

Serial Number	Name of the Publishers	Total Number of Journal Articles
1	American Chemical Society	3323
2	Wiley	3026
3	Royal Society of Chemistry	2140
4	Elsevier	8790
5	Springer	2668
6	Taylor & Francis	299
**Total**		**19,846**
